# The Subject, Its Biology, and the Chronic Recurrent Cystitis

**DOI:** 10.1155/2012/601705

**Published:** 2012-04-18

**Authors:** Michael Noll-Hussong, Michael Autenrieth, Dan Pokorny, Simone Herberger, Dorothea Huber

**Affiliations:** ^1^Clinic for Psychosomatic Medicine and Psychotherapy, University of Ulm, Am Hochstraess 8, 89081 Ulm, Germany; ^2^Klinik und Poliklinik fuer Psychosomatische Medizin und Psychotherapie, Technische Universitaet Muenchen, Langerstrasse 3, 81675 Muenchen, Germany; ^3^Urologische Klinik und Poliklinik des Klinikums rechts der Isar, Technische Universitaet Muenchen, Ismaninger Strasse 22, 81675 Muenchen, Germany; ^4^Klinik fuer Psychosomatische Medizin und Psychotherapie, Klinikum Harlaching, Staedtisches Klinikum Muenchen, Sanatoriumsplatz 2, 81545 Muenchen, Germany; ^5^International Psychoanalytic University Berlin, Stromstrasse 3, 10555 Berlin, Germany

## Abstract

Functional disorders in urology are troubling for both patients and physicians. Moreover, advances in recent research promise to provide biological insights into psycho-neuro-endocrino-immunological pathways that are one important facet of chronic urogenital inflammations. We present a case of a middle-aged woman with long-lasting recurrent cystitis for which especially a psychosomatic approach helped to understand and cure the disorder. Altogether, as practitioners treat subjects, not illnesses, a biopsychosocial understanding of human disease should be taken into account in cases of chronic recurrent cystitis.

## 1. Introduction

Somatoform disorders in urology are troubling for both patients and physicians [1]. The diagnosis relies on the presence of subjective distress in the absence of objective medical findings [2, 3]. As a result, there is the possibility that a diagnosis might be “missed.” A clear underlying physiology of distress exists, which implies that there is a two-way street, both psychosomatic and somatopsychic, in terms of the production and experience of functional urogenital symptoms [4, 5]. Studies on communication pathways from the immune system to the brain have presented exciting new research on the pathophysiology of the inflammation-associated symptoms of somatoform disorders [6].

The results from neuroimaging studies confirm the significance of the alterations of stress regulation [7], catastrophizing [8–10], and coping in somatoform pain disorders [11], along with the relevance of a history of physical or sexual abuse in the functional disorders studied thus far [12, 13]. Not only does the measurement of inflammation using biomarkers clinically document relevant infection, biomarkers also function as important tools to pinpoint the potentially harmful effects of chronic psychosocial stressors, such as job-related stress, low socioeconomic status, and childhood adversities [14, 15], as well as other life events, such as caregiver stress and loneliness [16, 17]. Similar results have been obtained in animal studies. For example, research has shown that early-life psychological stress exacerbates adult mouse asthma via the hypothalamus-pituitary-adrenal axis [18].

Cystitis is an inflammation of the bladder. It is usually assumed that this inflammation is caused by a urinary tract infection [19] in which the immunological preconditions remain unclear. Typical symptoms include pain when passing urine and frequent urination. Patients may also have pain in the lower abdomen, blood in the urine, and fever. Urine may become cloudy or smell unpleasant. Lower urinary tract infections are rare in males with normal urinary tracts [20]. On the other hand, about half of all women have at least one bout of cystitis in their life. Many women will only suffer from one or two bouts in their lifetime. However, some women suffer from recurring bouts of cystitis. This most commonly occurs in women who are in their late 20s or over the age of 55 and probably does not warrant an anatomic investigation. Cystitis definitely causes discomfort and inconvenience, but it is not life threatening and seldom leads to permanent damage of the urinary tract. Treatment is primarily aimed at the alleviation of symptoms. In most cases, there is no apparent reason why cystitis recurs [21, 22]. When recurrent cystitis [23, 24] poses a problem, antibiotic therapy may be indicated. The psychosomatic aspects of chronic recurrent cystitis have only rarely been the subject of (case) reports [25].

## 2. Case Report

### 2.1. Anamnesis

A 52-year-old housewife had been suffering from recurrent cystitis for at least 12 years. Over the last year, she took “a different antibiotic drug every month” and additionally received repeated “bladder flushes.” Yearlong urological treatments, as well as internal and gynaecological examinations, had not produced an organic finding that adequately explained the symptoms, nor had the treatments achieved lasting therapeutic success. At times, the symptoms were “determined” to be a bacterial pollakiuria and dysuria accompanied by evidence of bacteria in the urine and vaginal swabs, which mostly consisted of *Escherichia coli* and *Enterococci*. The urological examination and urodynamics did not reveal any irregularities other than a hypertonic bladder. A subsequent meatotomy did not improve the symptoms. In the acute stage, the patient suffered from pollakiuria, terminal searing, recurrent macrohematuria and constant bladder pressure with dysuria. 

The patient claimed to have suffered from recurrent depression since puberty, which specifically occurred in threshold situations (e.g., during the one-month absence of her mother during adolescence or the start of her university studies) and during the winter. Depression had, however, only been diagnosed at the age of 48 and resulted in behaviour therapy, which the patient ended after 50 hours. The temporary amelioration of the depression had “not been stable,” and antidepressants were therefore prescribed. According to the family medical history, the mother had also suffered from depression.

#### 2.1.1. Urological Findings

 Urine cultures (urine collected from a catheter) would sometimes be sterile and sometimes show the presence of *Escherichia coli*. The sonography revealed no irregularities of the bladder and kidneys (ambilateral) following urination (without the presence of any residual urine). Transvaginal urethrocystoscopy showed an inconspicuous urethral meatus. There was no evidence of a cystocoele with an inconspicuous urethra, and the mucous membrane of the urinary bladder, and the orifices were located in their normal anatomic (orthotopic) positions. The micturating cystourethrography showed a regularly configured bladder without evidence of vesicoureteral reflux (ambilateral). The excretory urogram revealed an inconspicuous pelvicalyceal system on both sides with unimpeded flow of contrast material through a delicate ureter. The abdominal and pelvic CT showed uterine fibroids, but there was no evidence of a rectovesical or a rectovesicovaginal fistula.

#### 2.1.2. Psychological Findings

 The gestures and facial expressions of the open-minded, highly motivated patient seemed clearly inhibited by a depressive affective state, albeit deflectable. A strong adaptability was evident, accompanied by a predominantly insecure personality with a compulsive defensive structure, particularly in terms of affective isolation. The level of the organization of the personality was considered high.

#### 2.1.3. Differential Diagnosis

 During the process of bacterial identification, it was believed that the bacteria originated from anal and vaginal contamination of the midstream urine sample, which was further substantiated when a urine sample collected from a catheter (with the same clinical symptoms) showed no pathological findings. It was therefore assumed that the patient suffered from a somatoform autonomic dysfunction of the urogenital tract in addition to a chronic recurrent bacterial cystitis, a finding that also corresponds with the psychological symptoms.

#### 2.1.4. Biography

Birth and early childhood development were normal. The patient had a carefree childhood up to the age of nine. She was then separated from her father, moved from a large city to the countryside, and lived in complete seclusion with her increasingly depressive mother. Her mother passed away when the patient was 16, at a time when she was experiencing severe emotional pain. Temporary failures at school followed. She met her husband one year after her mother's death and valued the “high degree of security” he gave her. She completed her university education, but she quit her job after the birth of her first child and began taking care of the household and caring for their four children. Her husband pursued his career. He had little time for her, and she felt neglected. At the time of the examination, she had not had a sexual relationship with her husband for a year. She “uses” her bladder as an excuse, since sexuality “is a risk factor for bladder infections.”

#### 2.1.5. Psychodynamics

Psychodynamically, the recurring bladder problems were closely linked to a partnership problem. She suppressed personal desires and needs, in particular aggressive impulses and individuation efforts, with rigid defence mechanisms. These suppressed affects and impulses seem to have shifted and manifested themselves as the affect equivalent of bladder problems. The organ of choice—and this was confirmed during the course of treatment—also indicated a sexual problem. In the context of a secondary gain from her illness, the patient only accepted her sexual denial to her husband under the cover of a somatic symptom. The simultaneous depressive symptoms can easily be explained by the mechanism of turning aggression against oneself. In addition, the patient had, to a great extent, unconsciously identified herself with her depressive mother, which was reinforced by an unconscious sense of guilt.

#### 2.1.6. Diagnosis

 Somatoform autonomic dysfunction of the urogenital system (ICD-10: F45.34); psychological factors in chronic recurrent cystitis (ICD-10: F54 at N30.2); recurrent depressive disorder, moderate episode (ICD-10: F33.1) [3].

#### 2.1.7. Behavioural Instruments

Patient therapy (see below) was controlled at the time points t1 (start of inpatient therapy), t2 (end of inpatient therapy), and t3 (follow-up 5 years later) using the Symptom Checklist-90-R (SCL-90-R) [26, 27].

According to the homepage of the publishing house Pearson Assessments [28], “the Symptom Checklist-90-R (SCL-90-R) instrument helps evaluate a broad range of psychological problems and symptoms of psychopathology. The instrument is also useful in measuring patient progress or treatment outcomes.” The 90 items of the checklist are scaled from 0 to 4 and are associated with the problems the patient has been suffering from during the last seven days. The summarizing global severity index (GSI) is a de facto standard for psychotherapy clinical practice and research, and serves as a kind of “symptom severity thermometer.” The nine specific subscales of the GSI provide an overview of the spectrum of patient complaints.

## 3. Therapy and Course of Treatment

In accordance with the psychotherapist's recommendations, the patient was referred to a psychodynamically oriented inpatient psychotherapeutic treatment centre. Initially, the patient's treatment focused on introjection as a psychological coping mechanism. The psychodynamics of suppressed aggression were also discussed with the patient. Her attitude as a child towards her dominant father, which in the form of transference also persisted in her marriage towards her husband, denoted the psychogenic background. Referring to the afflicted cloaca organ of choice, the patient addressed both sexual and passive-aggressive problems during treatment. The extent to which sexuality was being subverted by her compulsive defense mechanisms and the fact that a power struggle had evolved in the couple's sexual relationship was discussed. An exchange between the two partners took place. Within the scope of the nine-week inpatient psychotherapy, the patient came to understand the psychological background of her symptoms and, initially, no longer suffered from any bladder-related problems. The patient continued taking antidepressant medication, and the symptoms of depression improved.

The patient formulated the goal to stabilize the achievements she had made in inpatient psychotherapy, which in her view were pronounced, by continuing outpatient treatment in the form of depth psychotherapy. Due to a renewed deterioration of symptoms since her release of inpatient treatment, such stabilization seemed quite urgent. Accordingly, the patient could make use of supportive techniques as well as confrontations and other approaches at external venues. She had, for the first time in her life, acknowledged her own vitality and had come to understand the extent to which she compulsively denied her partner this vitality. The patient also understood the implications of her masochism for interpersonal relationships. She had always believed that she could only gain recognition by, or derive power from, assuming the role of the victim in her family. Her depressive mother often sought closeness to and comfort from the patient, yet she could not provide her with any security.

Through psychotherapy, she now has the capacity to construct corrective emotional experiences. She has coined the term “handbag therapist,” because she always carries a part of her relationship with the therapist in her handbag. She has been pursuing independent leisure activities for the first time in her life, and she has also attended training courses such as learning the French language and to use a personal computer. Her dreams (“falling into an abyss”; “falling into dark opaque water”; “having to care for a sick child at risk”) have been associated with the fear that her need for independence and the steps she is taking to achieve it creates. Since completing the approved 80-hour depth psychotherapy within the scope of German guideline psychotherapy [29], the patient continues to feel the need for additional low frequency sessions.

While bladder problems repeatedly flared up in the first year of therapy, the patient has not suffered from any urogenital symptoms in the following years. Her depression temporarily increased when the physical symptoms ceased, yet at the end of her psychotherapy, the patient was able to gradually decrease the intake of antidepressants. 

**Figure 1:**
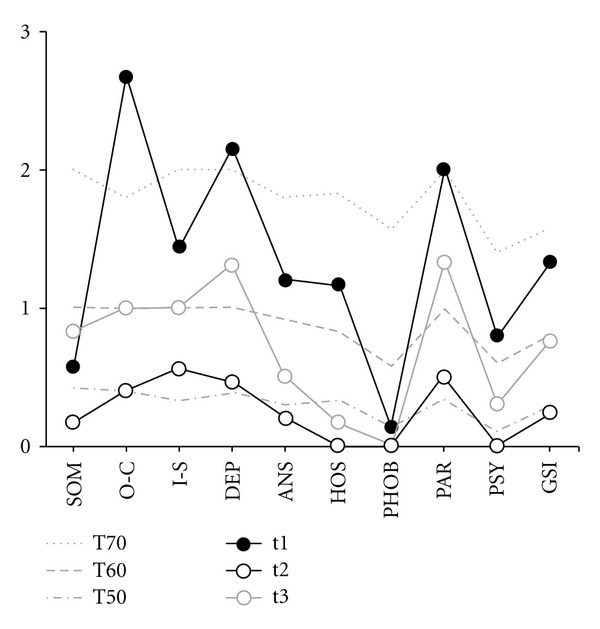
Illustration of the patient's self-assessment using the symptoms checklist SCL-90 R at 3 measurement points: start of inpatient therapy (t1), end of inpatient therapy (t2), and follow-up (t3). All 9 subscales (SOM: somatization, O-C: obsessive-compulsive, I-S: interpersonal sensitivity, DEP: depression, ANS: anxiety, HOS: hostility, PHOB: phobic anxiety, PAR: paranoid ideation, and PSY: psychoticism) and the GSI (Global Severity Index) are presented. Specifically, the subscales of compulsiveness, depression, and paranoia are elevated. At the end of therapy, the patient's values lie very close to the mean values of the healthy population in all scales. While the subscales of depression and paranoia have slightly increased again at the end of her treatment, they remain far below the initial values. (T50, T60, and T70: thresholds for the standard female German population. T60 is considered as “clinically suspicious” and T70 as “clinically relevant.” For the GSI, the T60 is already considered as “clinically relevant”).

We can now look back at the five-year follow-up period during which the patient has not suffered from any functional symptoms. There has been no recurrence of the cystitis and no shift to other organs.  Figure 1 shows the impressive decrease from the clinically pathological area (t1) to the values around the center (median) of the general population (t2). There was consistent improvement across the GSI as well as on all nine particular scales (all 90 items were valued by 0 or 1 (no problems in fact) at t2 (Table 1)). During follow-up (t3), a partial recurrence of some symptoms was observed, although the effect of these symptoms was considerably less than at intake (t1). The patient still showed improvement both on the GSI and on eight subscales (Figure 1). A comparison between follow-up (t3) and the initiation of therapy (t1) showed that 37 items were still improved and 8 worsened. The overall balance remained clearly positive (Table 1).

## 4. Discussion

The paradigms for the pathogenesis of urinary tract infections [30, 31] have shifted dramatically as a result of ongoing scientific revelations [20]. Beyond extracellular colonization of the bladder luminal surface, as traditional clinical thinking would hold, uropathogenic bacteria like *Escherichia coli* [32] direct a complex, intracellular cascade that shelters bacteria from host defences and leads to persistent bacterial residence within the epithelium. Following epithelial invasion, many organisms are promptly expelled by the epithelial cells of the bladder; a minority establish a niche in the cytoplasm that results in the development of biofilm-like intracellular bacterial communities that serve as the primary location for bacterial expansion. Exfoliation of the superficial epithelial layer may reduce the bacterial load, but it also facilitates the chronic residence of small nests of bacteria that later reemerge to cause some episodes of recurrent cystitis, a familiar clinical scenario in otherwise healthy women. Advances in both in vitro and animal models of cystitis [33] promise to provide biological insights into psycho-neuro-endocrino-immunological pathways [34] that are one important facet of this urogenital disease [35]. Furthermore, there seems to be a genetic component for increased susceptibility to such inflammations [36]. Diverse, predominantly somatic procedures [37] have been used to attempt to “cure” this disease (e.g., immunomodulatory approaches [31] and acupuncture [38]); however, there has been no larger evidence-based research that convincingly shows the effectiveness of these approaches. The basis of the diagnostic work-up of recurrent cystitis involves first the compilation of a precise medical history to be used in conjunction with the known pathogenesis of urinary tract infections [39, 40]. The anamnesis should also focus on factors that influence the state of the natural flora (sexual intercourse and hygiene) along with factors such as the history of previous antibiotic treatment and diseases that affect the immune status (like diabetes mellitus and psychological distress). A urinalysis is still the principal diagnostic procedure. The immediate analysis of a clean catch midstream urine sample, using a counting chamber or a test strip [41], promptly confirms the diagnosis and is then followed by a microbiological analysis and diagnosis.

Extended—but not necessarily repetitive—diagnostic work-up (urological staging) is aimed at detecting functional and anatomic abnormalities. While these factors only play a subordinate role during the premenopausal phase [42, 43], they become increasingly important during the postmenopausal phase. A key role is also attributed to local estrogen deficiency [44].

Interestingly, symptomatic and asymptomatic bacteriuria is common in pregnant women as well. A history of previous urinary tract infections and low socioeconomic status are risk factors for bacteriuria in pregnancy. *Escherichia coli* is the most common aetiologic agent in both symptomatic and asymptomatic infections, and a quantitative culture is the “gold standard” for diagnosis. Treatment of asymptomatic bacteriuria has been shown to reduce the rate of pyelonephritis in pregnancy. The screening for, and treatment of, asymptomatic bacteriuria has now become a standard of obstetrical care. The antibiotic treatment of asymptomatic bacteriuria is associated with a decrease in the incidence of low birth weight infants, but the methodological quality of the available studies limits the strength of their conclusions. Moreover, there is a debate in the literature as to whether treated pyelonephritis is associated with adverse foetal outcomes. There is no clear consensus in the literature on the choice of the antibiotic or the duration of the therapy in the treatment of the infection [45].

Using both psychometric questionnaires and psychiatric interviews, a study more than 30 years ago showed that women with recurrent cystitis have significantly more psychiatric symptoms (particularly anxiety) than the population as a whole. The study group showed a 3-fold increase in psychiatric symptoms antedating micturition symptoms and a 10-fold increase in psychiatric symptoms overall. Significant differences in the psychometric profiles have been shown between the different clinical subgroups of patients (e.g., bladder instability, outlet obstruction, and dyspareunia). A multifactorial approach (including an awareness of psychiatric factors) for patients suffering from recurrent cystitis can produce a treatment failure rate as low as 4.4% [46].

Treating patients with somatic and psychological symptoms calls for careful anamnestic exploration and a thorough clinical examination [47]. The current dualistic [48] classification of both functional (somatic) and somatoform (psychological) disorders [2, 3] is currently being revised in order to improve its validity for the DSM-V and ICD-11 [49–54] and should take into account evolving neurobiological insights [55]. The incorporation of a dimensional approach that reflects both somatic and psychological symptoms severity also has the potential to improve predictive validity and clinical utility [56, 57]. By providing new approaches to cope with distressing events, multimodal psychodynamic psychotherapy has the power to decrease symptoms, influence the immune system, and normalize neuronal activity [58].

## Figures and Tables

**Table 1:** Severe symptom items (value 3 or 4 at any time point) of the SCL-90 R. Seventeen items the patient highly complained of at t1 (value 3 or 4) are presented. (t1: beginning of inpatient therapy. t2: end of inpatient therapy. t3: follow-up. The items are organized by corresponding scales. The scales are sorted by their value at the intake).

Scale/item	t1	t2	t3
Obsessive-compulsive			
Having to do things very slowly to insure correctness	4	1	2
Repeated unpleasant thoughts that won't leave your mind	4	0	2
Trouble concentrating	3	1	2
Your mind going blank	3	0	1
Having to check and double-check what you do	3	0	0
Depression			
Feeling everything is an effort	4	1	3
Loss of sexual interest or pleasure	4	1	3
Worrying too much about things	4	0	2
Feeling low in energy or slowed down	3	1	3
Feeling lonely	3	1	1
Feeling blue	3	1	0
Paranoid ideation			
Others not giving you proper credit for your achievements	3	0	2
Feeling others are to blame for most of your troubles	3	0	0
Interpersonal sensitivity			
Your feelings being easily hurt	3	1	2
Feeling critical of others	3	1	2
Feeling others do not understand you or are unsympathetic	3	1	1
Anxiety			
Feeling fearful	4	1	0
Feeling tense or keyed up	3	1	2
Hostility			
Feeling easily annoyed or irritated	3	0	1
Complementary items			
Sleep that is restless or disturbed	3	0	2
Awakening in the early morning	3	0	1
Psychoticism			
The idea that something serious is wrong with your body	4	0	0
